# Left Ventricular Pseudoaneurysm With a Bead-Like Appearance After Myocardial Infarction: The Utility of Cardiac Computed Tomography

**DOI:** 10.7759/cureus.22773

**Published:** 2022-03-02

**Authors:** Shinsuke Kido, Tetsuya Kosaki, Akinori Higaki, Hiroshi Ishitoya, Hideki Okayama

**Affiliations:** 1 Department of Cardiology, Ehime Prefectural Central Hospital, Matsuyama, JPN; 2 Department of Cardiovascular Surgery, Ehime Prefectural Central Hospital, Matsuyama, JPN

**Keywords:** aneurysmectomy, ct coronary angiography, adult cardiac surgery, myocardial infarction, left ventricular pseudoaneurysm

## Abstract

A left ventricular pseudoaneurysm is a rare but life-threatening complication after myocardial infarction. Because untreated pseudoaneurysms have a 30%-45% risk of rupture, surgery is the preferred therapeutic option. However, its diagnosis is sometimes challenging, as a pseudoaneurysm presents with non-specific symptoms that can mimic myocardial infarction or heart failure. We report a male patient with a history of aortic dissection surgery who presented with recurrent chest pain probably due to acute coronary syndrome. Transthoracic echocardiography revealed a cavity at the apex of the left ventricle, indicating a mechanical complication after myocardial infarction. As the coronary angiography was considered difficult because of the patient’s anatomical problem, contrast-enhanced computed tomography (CT) was performed. CT angiography revealed multiple nodular cavities continued from within the left ventricle. It seemed that the pseudoaneurysm was formed in stages in the adherent pericardium after myocardial infarction, resulting in a bead-like appearance. Emergent pseudoaneurysmectomy and left ventricular wall repair were performed, and the patient was discharged without any complications. This case illustrates the utility of cardiac CT to establish the diagnosis of left ventricular pseudoaneurysm and coronary artery atherosclerosis.

## Introduction

A left ventricular pseudoaneurysm is a rare but life-threatening complication after myocardial infarction. Because untreated pseudoaneurysms have a 30%-45% risk of rupture, early diagnosis and surgical repair are required [1-2]. However, its diagnosis is sometimes challenging if the pseudoaneurysm is asymptomatic or exhibits a rare morphology [3]. We report a case of a patient who developed a left ventricular pseudoaneurysm with a bead-like morphology, possibly due to a history of prior cardiac surgery.

## Case presentation

**Figure 1:**
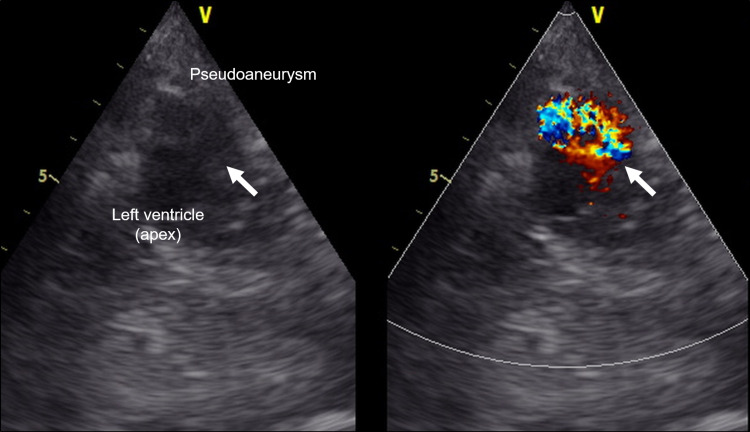
Transthoracic echocardiography taken at an emergency department A short-axis view of transthoracic echocardiography revealed a cavity at the apex. Color Doppler imaging showed abnormal blood flow from the left ventricle toward the cavity. The arrows indicate the orifice of the pseudoaneurysm.

A 67-year-old man with a history of aortic dissection surgery (a hemiarch replacement to the aortic root) was transferred to our hospital by his family doctor with a tentative diagnosis of acute myocardial infarction. He had experienced chest pain two weeks before being referred to our hospital, but he did not visit his home doctor, as his symptoms improved spontaneously. Physical examination revealed his heart rate was 129 beats per minute, his arterial blood pressure was 168/66 mmHg, and peripheral oxygen saturation was 98% while breathing ambient air. On auscultation, a to-and-fro murmur (during both systolic and diastolic) was heard on the cardiac apex. His electrocardiogram showed an elevated ST-segment in the V1-4 leads with a reduced R wave height. Transthoracic echocardiography revealed a cavity at the apex and color Doppler imaging revealed an abnormal blood flow from the left ventricle toward the cavity (Figure 1), and minimal pericardial effusion.

**Figure 2:**
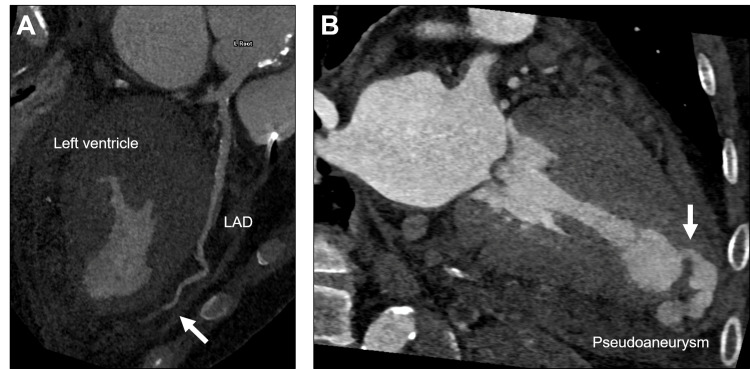
The preoperative cardiac computed tomography image Panel A shows a curved planar reconstruction image showing severe stenosis in the distal part of the left anterior descending artery indicated by the arrow. Panel B shows a long-axis view of the left ventricle showing pseudoaneurysms with a bead-like appearance. The arrow indicates the orifice of the pseudoaneurysm.

At this point, the mechanical complications of myocardial infarction were suspected, and the use of circulatory support devices and coronary angiography was decided. However, the patient had graft anastomosis to the ascending aorta performed during the total arch replacement and strong tortuosity of the brachiocephalic artery due to the prior surgery for the aortic dissection. In addition, he had a shunt in the left arm for dialysis, and dissection in the descending aorta, which made an approach from the upper and lower limbs for coronary angiography difficult. Therefore, we performed cardiac computed tomography (CT) with contrast enhancement to obtain anatomical vascular and coronary information. Cardiac CT showed severe stenosis in the distal part of the left anterior descending artery (LAD) and a pseudoaneurysm communicating with the apical left ventricular cavity through a narrow neck (Figure 2).

**Figure 3:**
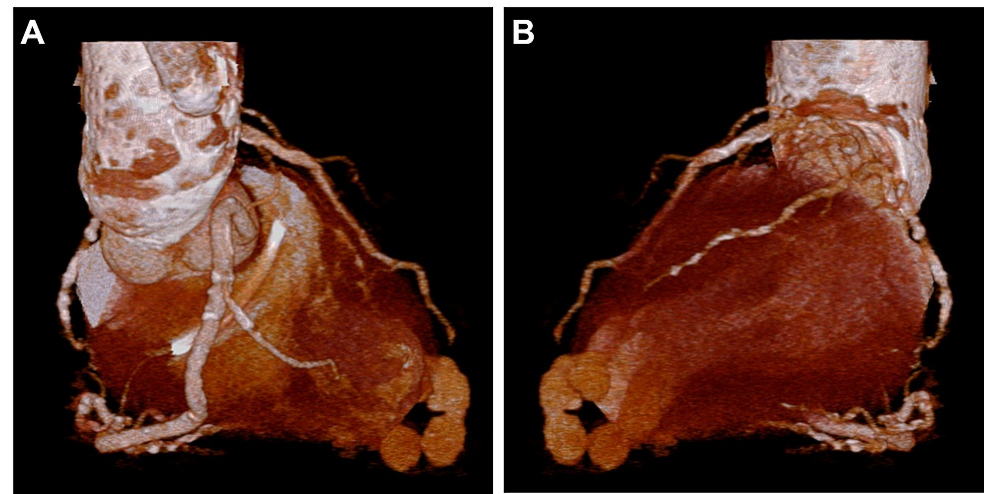
Three-dimensional computed tomography Three-dimensional volume-rendering images clarified the morphology of the pseudoaneurysm. Left anterior oblique 50 (panel A) and 130 (panel B) images show a spiral sequence of pseudoaneurysms starting at the left ventricular apex, resulting in a bead-like appearance.

Three-dimensional volume-rendering images revealed a spiral sequence of pseudoaneurysms starting at the left ventricular apex, resulting in a bead-like appearance (Figure 3).

**Figure 4:**
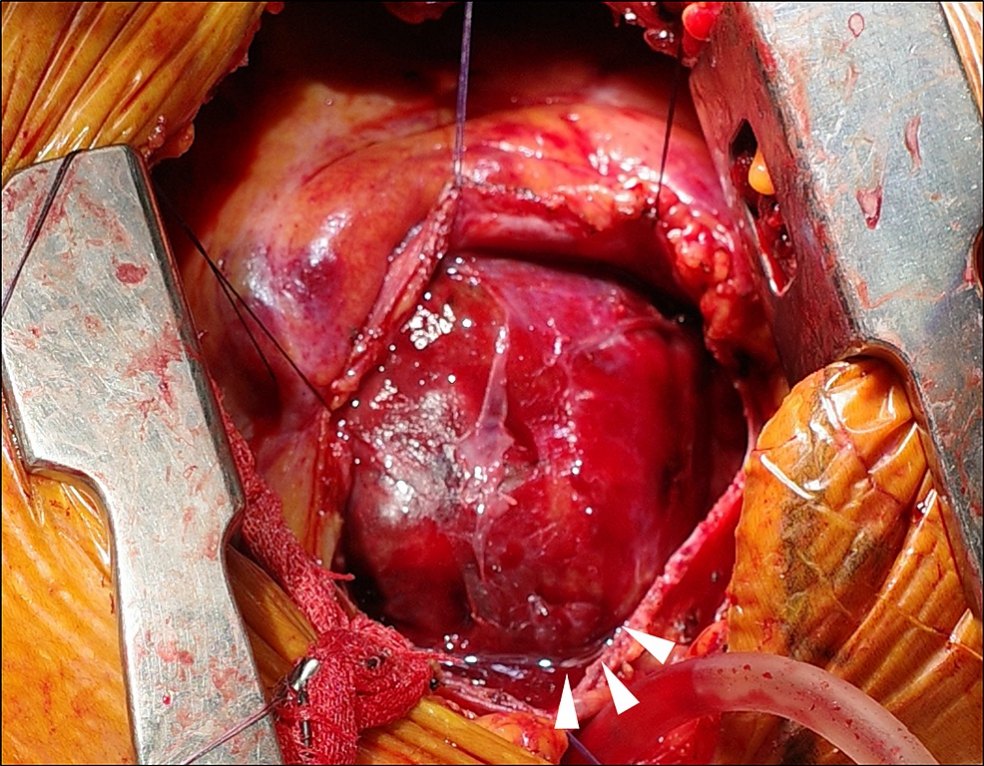
Intraoperative findings When the pericardium was cut open, the pseudoaneurysm was present in the pericardial cavity as a hematoma in contact with the myocardium (arrowheads).

After discussing treatment options with the cardiovascular surgeon, emergent pseudoaneurysmectomy and left ventricular repair were performed. Coronary artery bypass graft was not performed since the site of the coronary artery occlusion was very distal to the LAD, and there was no significant stenosis in the other coronary arteries. Since the vascular graft was located just under the sternum and clamping of the central site of the brachiocephalic artery was expected to be difficult, we selected a left thoracotomy rather than a midline sternal incision. When the pericardium was cut open, the pseudoaneurysm was present in the pericardial cavity as a hematoma in contact with the myocardium (Figure 4). Incision of the hematoma revealed an 8-mm rupture hole in the left ventricular wall, which was repaired with felted sutures. The patient’s postoperative course was good, and he was discharged 23 days after the surgery without any complications.

## Discussion

Left ventricular pseudoaneurysms are formed when cardiac rupture is contained by adherent pericardium [1]. A true aneurysm involves all three layers of the arterial wall that enlarge together while a pseudoaneurysm involves a persistent communication between the vessel and the surrounding tissue cavity. The pseudoaneurysm will typically have a narrow orifice with a single chamber, but its shape and size can be affected by the degree of pericardial adhesion [4-5]. Roa-Castro and colleagues reported a case with a pseudoaneurysm after myocardial infarction in which the dense pericardial adhesion after coronary artery bypass surgery might prevent the fatal outcome of free wall rupture [6]. There are also several case reports suggesting dense pericardial adhesion secondary to previous cardiac operation prevented traumatic right ventricular rupture [7-8]. However, there have been no previous reports of pseudoaneurysms with a bead-like shape in the left ventricular apex after surgery for aortic dissection. In our case, color Doppler echocardiography was the first clue to the diagnosis. The color Doppler depicted abnormal blood flow from the left ventricle toward the cavity, suggesting the presence of the pseudoaneurysm. It has been reported that two-dimensional echocardiography may aid in the diagnosis, but it does not always lead to a definitive diagnosis in all cases [1].

Cardiac contrast-enhanced CT allows for three-dimensional morphological evaluation [2] and is very useful, especially when access for invasive angiography is limited, as in this case. It seemed that the left ventricular pseudoaneurysm was formed in stages in the adherent pericardium, which fortunately allowed the patient to visit the hospital without developing a blowout cardiac rupture or severe cardiac tamponade. However, if the disease had progressed one step further, the patient would have fallen into a serious condition, and a quick differential diagnosis was necessary to determine the indication for surgery. As a differential diagnosis on imaging, a coronary artery fistula may show a similar nodular morphology [9]. In this case, there was no connection between the cavity and the coronary artery, which allowed us to differentiate it from a fistula.

It remains unclear what patient factors lead to the development of pseudoaneurysms in the form of beads. As the degree of pericardial adhesion may vary depending on the type of cardiac surgery and patient factors, further research is warranted to confirm the process of pseudoaneurysm formation.

## Conclusions

In patients with prior cardiac surgery, left ventricular pseudoaneurysms after myocardial infarction can exhibit a bead-like morphology, possibly due to the highly adhesive pericardium. Cardiac CT was useful to establish the diagnosis of left ventricular pseudoaneurysm and coronary atherosclerosis, especially when the access site for invasive angiography is limited.
